# Seroepidemiological study of *Leishmania infantum*, *Toxoplasma gondii* and *Dirofilaria immitis* in pet ferrets (*Mustela putorius furo*) in Spain

**DOI:** 10.1007/s11259-025-10729-5

**Published:** 2025-04-07

**Authors:** José Villora, María Eugenia Lebrero, Jacobo Giner, Asier Basurco, Laura Vilalta, Andrés Montesinos, Maria-Magdalena Alcover, Cristina Riera, Roser Fisa, Xavier Roca-Geronès, Aitor Ramos, Álex Gómez, Sergio Villanueva-Saz, Antonio Fernández, Diana Marteles

**Affiliations:** 1 Selvática VetPartners Veterinary Clinic, Valencia, Spain; 2 Department of Animal Pathology, Universidad de Zaragoza, 177 Miguel Servet Street, Zaragoza, 50013 Spain; 3 Menescalia Veterinary Clinic, Ismael Merlo Actor, Valencia, Spain; 4 Centro Médico Veterinario Maidagan, Getxo, Spain; 5 Hospital Veterinari Canis, Girona, Spain; 6 Hospital Veterinario de Animales Exoticos Los Sauces, Madrid, Spain; 7 Department of Animal Medicine and Surgery, Universidad Complutense de Madrid, Madrid, Spain; 8 Department of Biology, Faculty of Pharmacy, Health and Environmental Medicine, Universitat de Barcelona, Barcelona, Spain; 9 Urgencias Veterinarias, Zaragoza, Spain; 10 Clinical Immunology Laboratory, Veterinary Faculty, Universidad de Zaragoza, Zaragoza, Spain; 11 Agro-food institute of Aragón-IA2 (University of Zaragoza-CITA), Universidad de Zaragoza, Zaragoza, Spain

**Keywords:** *Dirofilaria immitis*, Ferret, *Leishmania infantum*, Serology, Spain, *Toxoplasma gondii*

## Abstract

Ferrets are susceptible to infections with *Leishmania infantum*, *Toxoplasma gondii*, and *Dirofilaria immitis*; however, epidemiological data on these infections are limited in Europe. A total of 448 serum samples from household ferrets were collected between December 2019 and December 2023 in Spain. In this study, we assessed the seroprevalence of *L. infantum* and *D. immitis* using an in-house enzyme-linked immunosorbent assay (ELISA) and *T. gondii* using an in-house immunofluorescence antibody test (IFAT). Among the ferrets tested, the seroprevalence was 10.49% (47/448) for *L. infantum*, 2.68% (12/448) for *T. gondii* and 10.27% (46/448) for *D. immitis*. There was no significant association between seropositivity and age, gender, neutering status, cohabitation, lifestyle, and collection date. Ferrets classified as sick animals related to the presence of compatible or non-compatible signs showed a higher seropositivity rate for *L. infantum* (15.90%) compared to subclinical animals (4.76%). Overall, 23.44% (105/448) of the samples were seropositive for at least one of the three parasitic agents, 3.12% (15/448) were positive for two agents, and 0.22% (1/448) tested positive for all three agents. Co-infections were also evaluated, revealing that 12.76% (6/47) of *L. infantum* seropositive ferrets were also positive for *T. gondii* (*p* = 0.011) and 21.27% (10/47) for *D. immitis* (*p* = 0.009). To the best of our knowledge, this is the first report on the seroprevalence of *L. infantum*, *D. immitis*, and *T. gondii* within the ferret population in Spain. Understanding the epidemiological status of these and other zoonotic pathogens is crucial for enhancing surveillance in both veterinary and public health sectors, as well as for strengthening prevention and control strategies.

**Supplementary Information:**

The online version contains supplementary material available at 10.1007/s11259-025-10729-5.

## Introduction

Ferrets (*Mustela putorius furo*), a small mustelid domesticated in Europe approximately 2000 years ago, are increasingly popular pets worldwide (Powers and Perpiñan 2021; D’Ovidio et al. 2014). In Spain, ownership between 2012 and 2015 increased exponentially, and according to the latest government data, Spain has an officially registered population of 20,000 pet ferrets (MAGRAMA 2015). However, the scientific information related to zoonotic parasites, such as the protozoans *L. infantum* and *T. gondii*, and the nematode *D. immitis* in ferrets in European countries is very limited.

Leishmaniosis is one of the most significant vector-borne diseases in Europe, with dogs serving as the primary domestic reservoir. Nevertheless, other animals such as cats, rabbits, and hares have also been implicated in leishmaniosis epidemiology and transmission (Alcover et al. 2020; Giner et al. 2022). Recently, the first clinical cases of seropositive, symptomatic ferrets were reported in Spain (Alcover et al. 2021; Giner et al. 2021; Villanueva-Saz et al. 2021). Clinical signs in ferrets with leishmaniosis can vary, with anorexia, peripheral lymphadenopathy, and dermatological lesions being the most common (Giner et al. 2024a, b). Laboratory findings often include anemia, thrombocytopenia, and hyperproteinemia characterized by hypergammaglobulinemia or hyperbetaglobulinemia (Giner et al. 2020). Confirmatory methods typically involve polymerase chain reaction (PCR) of blood, skin or bone marrow samples (Villanueva-Saz et al. 2021) and quantitative serology (Reis et al. 2006; Alcover et al. 2022; Solano-Gallego et al. 2011). The seroprevalence of *L. infantum* in ferrets from Spain was reported to be 28.4% (Alcover et al. 2022). This contrasts with previous studies in other animal species, such as the European mink (*Mustela lutreola*) and the American mink (*Neovison vison*), which showed seropositivity rates of 45.3% and 52.4%, respectively (Giner et al. 2022). Further epidemiological studies are required in Spain, extending beyond the Mediterranean basin, to better understand the epidemiological role of domestic ferrets in *L. infantum*-endemic areas.

Domestic cats and other felids serve as the primary hosts for *T. gondii* (Davidson 2000). Exposure to *T. gondii* has been documented in multiple mustelid species, including *Neovison vison*,* Lontra canadensis*, *Martes americana*, and *Martes pennanti* (Tizard et al. 1976; O’Crowley and Wilson 1991; Tocidlowski et al. 1997; Martino et al. 2017). Ferrets may act as intermediate hosts, with clinical toxoplasmosis manifesting as ocular lesions, jaundice, neurological symptoms, diarrhea, lethargy, anorexia, respiratory distress, and fever (Lescano and Quevedo 2017; Powers 2009). Confirmatory diagnosis of *T. gondii* involves a combination of serological and molecular techniques from affected tissues, such as blood or biopsied organs (Wyrosdick and Schaefer 2015). No seroepidemiological data on *T. gondii* in ferrets is currently available for Europe.

*Dirofilaria immitis*, responsible for heartworm disease, is transmitted by mosquitoes during blood feeding (McCall et al. 2008). While dogs are the primary reservoir of infection, ferrets are highly susceptible, with clinical presentations, including dullness, anorexia, coughing, dyspnea, heart murmur, pleural effusion, ascites, anemia, hemolysis, and acute renal or hepatic failure (Konder 2018; Morrisey and Malakoff 2021). Diagnostic methods for *D. immitis* detection in ferrets include molecular assays and echocardiography (Villanueva-Saz et al. 2022b). Indirect in-house ELISA tests using somatic antigens based on third-stage larvae (L3) detect specific antibodies as early as one month post-infection (Prieto et al. 2001), in comparison to other molecular assays such as PCR or direct ELISA, considered early diagnostic methods. A previous study of 186 domestic ferrets in Valencia, Spain, revealed a seroprevalence of 14.51% (Villanueva-Saz et al. 2022b).

Due to the limited epidemiological data on *L. infantum*, *T. gondii*, and *D. immitis* infections in pet ferrets in Spain, this study aims to improve understanding of the role of ferrets in these parasitic diseases. Specifically, we evaluate the seroprevalence of these infections across various regions of Spain and identify associated risk factors.

## Materials and methods

### Serum samples

Between December 2019 and December 2023, veterinarians from 16 provinces across Spain collected serum samples from 448 ferrets (*Mustela putorius furo*). The ferrets were randomly included in the study, based on their owners’ consent to use stored (−20 °C) serum samples. Additionally, detailed information was obtained on age (juvenile (≤ 1 year), adult (> 1 to < 5 years), or senior (≥ 5 years)), gender, neutering status (intact, surgically neutered, or hormone-implanted), cohabitation with other animals, clinical health status (suspect vs. non-suspect), lifestyle (indoor or mixed), use of ectoparasiticides and macrocyclic lactones, and geographic location. The date of serum collection was also recorded. Fifty serum samples lacked associated information about the ferrets. The geographical distribution was as follows: 128 ferrets from Madrid (31.1%), 106 from Valencia (25.8%), 56 from Zaragoza (13.7%), 32 from Girona (7.8%), 19 from Gran Canaria (4.6%), 18 from Álava (4.4%), 13 from Bizkaia (3.2%), 8 from Pontevedra (1.8%), 7 from Sevilla (1.7%), 6 from Alicante (1.5%), 5 each from Navarra (1.2%) and Murcia (1.2%), and 2 each from Burgos (0.5%), Huelva (0.5%), Castellón (0.5%), and Barcelona (0.5%) (Table 1).

**Table 1:** Geographical distribution of the sampled ferrets and seropositive cases

Provinces	Sample size (*n*/total population %)	L. infantum (*n* positive/%)	T. gondii (*n* positive/%)	D. immitis (*n* positive/%)
Madrid	128 (31.1)	19 (14.8)	4 (3.1)	9 (7)
Valencia	106 (25.8)	20 (18.9)	7 (6.6)	15 (14.2)
Zaragoza	56 (13.7)	1 (1.8)	1 (1.8)	10 (17.9)
Girona	32 (7.8)	1 (3.1)		2 (6.3)
Gran Canaria	19 (4.6)			1 (5.3)
Álava	18 (4.4)			1 (5.6)
Bizkaia	13 (3.2)			2 (15.4)
Pontevedra	8 (1.8)			
Sevilla	7 (1.7)			1 (14.3)
Alicante	6 (1.5)	4 (66.7)		1
Navarra	5(1.2)			1 (20)
Murcia	5 (1.2)	1 (20)		1 (20)
Burgos	2 (0.5)			
Huelva	2 (0.5)	1 (50)		
Castellón	2 (0.5)			1 (50)
Barcelona	2 (0.5)			1 (50)
Total	448	47 (10.49)	12 (2.68)	46 (10.27)

## Leishmania infantum antibodies

To detect *L. infantum*-specific antibodies, an in-house ELISA (99.37% sensitivity and 97.50% specificity) (Basurco et al. 2020), based on *L. infantum* antigen (MHOM/FR/78/LEM75 zymodeme MON-1), was performed as previously described, with modifications (Giner et al. 2020; Alcover et al. 2022). Briefly, 100 µL of serum, diluted 1:200 in phosphate-buffered saline (PBS) with 0.05% Tween 20 (PBST) and 1% skimmed milk (PBST-M), was added to each well, and plates were incubated for 1 h at 37 °C in a humid chamber. After washing twice with PBS, 100 µL of horseradish peroxidase-conjugated protein A, diluted 1:8000 in PBST-M, was added. Plates were incubated again for 1 h at 37 °C, washed with PBST and PBS, and incubated with ortho-phenylenediamine and a stable peroxide substrate buffer for 20 ± 5 min at room temperature in the dark. The reaction was stopped by adding 2.5 M H₂SO₄. Absorbance was read at 492 nm with an automatic ELISA reader (Multiskan ELISA reader, Labsystems, Midland, Canada). Each plate included positive control serum from a ferret with confirmed leishmaniosis and negative control serum from a healthy ferret. The cut-off value was set at an optical density (OD) of 0.180 using sera from 30 indoor ferrets from northern Spain (Formula used: mean of OD values + (3 x standard deviation of OD values). Values above this threshold were considered positive. Previous studies have shown that sonicated *L. infantum* antigens (MHOM/FR/78/LEM75 zymodeme MON-1) are highly specific in dogs, ferrets and minks (Giner et al. 2020; Alcover et al. 2022; Villanueva-Saz et al. 2021, 2022c), avoiding cross-reactivity with *T. gondii*.

## Toxoplasma gondii antibodies

An in-house IFAT (98% sensitivity and 96% specificity in the internal protocol of the laboratory) was conducted as a reference test using *T. gondii* formalin-fixed tachyzoites (Goldman 1957; Villanueva-Saz et al. 2023). Serum samples were diluted 1:32 in PBS, and 20 µL of each dilution was applied to the wells. Slides were incubated for 30 min at 37 °C in a humid chamber, then washed twice with PBS and once with distilled water. After washing, 20 µL of goat anti-Protein A fluorescein isothiocyanate conjugate, diluted 1:32 with 0.2% Evans blue, was added to each well (Davidson 2000). After a 30-minute incubation at 37 °C in the dark, slides were rewashed, mounted with medium, and examined under a fluorescence microscope (Leica DM750 RH, Berlin, Germany) at 400× magnification. Fluorescence patterns were compared to positive and negative controls. The positive control included serum from a *T. gondii*-infected cat and a *T. gondii*-infected ferret, while the negative control was from a healthy, non-infected ferret. A cut-off titer of 1:32 was used, with endpoint titers categorized as low (≥ 1:32 and ≤ 1:128), moderate (≥ 1:256 and ≤ 1:512), and high (> 1:512).

## Dirofilaria immitis antibodies

All sera were tested using an indirect ELISA, based on the pepsin inhibitor Dit33 (DIT33) recombinant protein of *D. immitis* (Urano Vet^®^, Barcelona, Spain). as described previously (Villanueva-Saz et al. 2022b). A 100 µL aliquot of serum, diluted 1:100 in PBS, was added to each well. Plates were incubated at room temperature in a humid chamber for 45 min, washed with PBST, and then treated with 100 µL of protein A-horseradish peroxidase conjugate, diluted 1:10,000 in PBST with 1% skimmed milk. Plates were incubated at 37 °C for 30 min, washed, and developed as described above. Absorbance was measured at 492 nm with an automatic ELISA reader (ELISA Reader Labsystems Multiskan, Midland, ON, Canada). Positive controls included serum from a *D. immitis*-infected ferret and negative controls from non-infected ferrets from non-endemic areas. The cut-off OD was set at 0.31 (mean OD ± 1 SD from non-infected, indoor ferrets from outside the study area), with values above 0.31 considered positive.

### Statistical analysis

Statistical analyses were performed using SPSS software, version 22.0 (IBM, Armonk, NY, USA), and WinEpi software. Descriptive analysis was conducted for the entire sample population, with exact binomial 95% confidence intervals (CIs) calculated for prevalence estimates. Univariate analyses of categorical variables (age, gender, neutering status, cohabitation, health status, lifestyle, and collection date) were performed to identify associations with seropositivity. Sera with no clinical information were excluded from this analysis. Associations were assessed for significance using Chi-square or Fisher’s exact tests, with a threshold of *p* ≤ 0.05. Sample sizes for each pathogen were calculated to achieve 95% CI, based on expected seroprevalence values: 9% for *L. infantum* (Davidson 2000), 14.51% for *D. immitis* (Villanueva-Saz et al. 2022b), and 30.56% for *T. gondii* (Cano-Terriza et al. 2016).

## Results

### Demographic structure of animals included in the study

A total of 64 ferrets (16%) were classified as juveniles, 154 (38.7%) as adults, and 180 (45.3%) as seniors. Among them, 180 (45.2%) were female, and 218 (54.8%) were male. Regarding reproductive status, 130 ferrets (32.5%) were intact, 162 (40.5%) were hormone-implanted, and 108 (27%) were surgically neutered. A total of 258 ferrets (70.9%) cohabited with other animals, while 106 ferrets (29.1%) lived alone. Lifestyle analysis showed that 258 ferrets (64.5%) had an indoor lifestyle, while 142 (35.5%) had a mixed lifestyle. None of the ferrets had been treated with ectoparasiticides and any ferret received macrocyclic lactones, not specifically for the prevention of *D. immitis*.

### *Seroprevalence of* Leishmania infantum

The overall seroprevalence of *L. infantum* was 10.49% (47/448). Among seropositive ferrets with available data, 24 were male and 22 females; 26 were seniors, 18 adults, and 2 juveniles (Supplementary Table S1). The geographical distribution of *L. infantum* seropositive ferrets included Valencia (20), Madrid (19), Alicante (4), and one each in Murcia, Girona, Zaragoza, and Huelva. Seropositivity was significantly associated with health status, with a higher prevalence among symptomatic ferrets (9.4%) compared to healthy ones (2%) (*p* = 0.02). There was no significant association between seropositivity and age, gender, neutering status, cohabitation, lifestyle, and collection date (*p* > 0.05).

### *Seroprevalence of* Toxoplasma gondii

The seroprevalence rate was 2.68% (12/448). Among seropositive individuals with epidemiological data, 4 were male and 5 females, with 7 classified as seniors and 2 as adults (Supplementary Table S1). Seropositive ferrets were found in Valencia (7), Madrid (4), and Zaragoza (1). There was no significant association between seropositivity and age, gender, neutering status, cohabitation, health status, lifestyle, and collection date (*p* > 0.05).

### *Seroprevalence of* Dirofilaria immitis

The seroprevalence of *D. immitis* was 10.27% (46/448), with 25 males and 20 females testing positive. Among those, 19 were seniors, 18 adults, and 8 juveniles (Supplementary Table S1). *D. immitis*-positive ferrets were distributed across provinces, including Valencia (15), Zaragoza (10), and Madrid (9), with sporadic cases in several other provinces. However, there was no significant association between seropositivity and age, gender, neutering status, cohabitation, health status, lifestyle, and collection date.

## Co-infections

Generally, 23.44% (105/448) were seropositive for at least one of the parasitic agents, 3.57% (16/448) were positive for two of the agents, and only 0.22% (1/448) tested seropositive for all three agents (Table 2). The prevalence of co-infections among the evaluated ferrets was 3.57% (16/448). The co-infection rate with *Leishmania* and one of the two additional tested pathogens was 34.04% (16/47). Significant co-infection rates were observed, with 12.76% (6/47) of *L. infantum* seropositive ferrets also positive for *T. gondii* (*p* = 0.011) and 21.27% (10/47) for *D. immitis* (*p* = 0.009). Only 2.1% (1/47) of *L. infantum* seropositive ferrets exhibited co-infection with both, *T. gondii*. and *D. immitis*.

**Table 2:** Ferrets’ infectious status based on *Dirofilaria immitis*, *Leishmania infantum* and *Toxoplasma gondii* serological test results

Dirofilaria immitis	Leishmania infantum	Toxoplasma gondii	Number of seropositive ferrets
-	+	-	30
+	-	-	35
-	-	+	5
-	+	+	6
+	+	-	10
+	+	+	1

## Discussion

*Leishmania infantum*, *T. gondii* and *D. immitis* are significant parasitic pathogens impacting various mammalian hosts worldwide, including companion animals such as dogs and cats. Recent research has also identified these parasites in ferrets (*Mustela putorius furo*) (Davidson 2000; Villanueva-Saz et al. 2022b) and other mustelids (Matsuda et al. 2003; Santoro et al. 2017; Penezić et al. 2018; Sengupta et al. 2021; Cotey et al. 2022; Ortuño et al. 2022; Upton et al. 2022; Alsarraf et al. 2023; Markakis et al. 2024). Despite this, information on the seroprevalence of these infections in pet ferrets remains scarce and largely anecdotal. This study is the first to investigate the presence of *L. infantum*, *T. gondii*, and *D. immitis* in ferrets in Spain. Overall, 23.44% of ferrets tested were seropositive for at least one of the three pathogens. The highest seroprevalence was found for *L. infantum* (10.49%), followed by *D. immitis* (10.27%), and *T. gondii* (2.68%). Seropositivity rates varied depending on the geographic region.

*Leishmania infantum* seropositivity was most prevalent in endemic regions of Spain, in line with previous findings reporting ferret *Leishmania* prevalence from 9% by ELISA to 25.5% by western blot (WB) in healthy ferrets (Alcover et al. 2022). In this study, the observed seroprevalence (10.49%) was lower than that reported in previous research conducted in Spain (Alcover et al. 2022). This difference is likely attributable to the geographic area of the studies and the serological technique used. The earlier study focused exclusively on the Comunitat Valenciana, a region highly endemic for *L. infantum*. In contrast, the present study included samples from 16 provinces, many of which exhibit a less severe endemic status for *L. infantum* compared to the Comunitat Valenciana. While WB demonstrated statistically higher sensitivity than ELISA, which would explain the lower prevalence result compared to that previously obtained using WB (Alcover et al. 2022), no significant difference in specificity was observed between the two tests. In addition, WB is typically used as a confirmatory test rather than a screening test, due to its higher sensitivity, technical complexity, and limited routine availability. Conversely, the ELISA technique has been proven to be suitable for detecting anti-Leishmania antibodies and monitoring their evolution over time in mustelids (Del Carmen Aranda et al. 2025).

Clinical leishmaniosis caused by *L. infantum* has been documented, with emerging reports of new dermatological forms (Giner et al. 2020, 2021, 2024a, b). Moreover, significant co-infection rates were observed, with *L. infantum* seropositive ferrets also positive for *T. gondii* or *D. immitis*. Therefore, these results suggest that immunosuppression from comorbid conditions could increase susceptibility to *L. infantum*.

*Toxoplasma gondii* seropositivity was observed in 2.68% of ferrets, representing the first documented prevalence in pet ferrets in Spain and Europe. Wild mink populations in Spain and other regions have shown higher seroprevalence (78.8% in Spain; 45.36% in Germany; 53.6% in Denmark) (Risueño et al. 2018; Sengupta et al. 2021; Heddergott et al. 2024). Clinical toxoplasmosis is rare in ferrets and usually associated with immunosuppression. No correlation was identified between seropositivity and lifestyle. This lack of association may be due to the fact that most ferrets are kept as pets, reducing their need to engage in natural hunting behaviors, and thus limiting their contact with potentially contaminated soil, even when housed outdoors. However, further research on ferret diet and exposure to cats could provide insights into transmission risk factors (Gauss et al. 2003; Villanueva-Saz et al. 2022a).

*Dirofilaria immitis* seroprevalence in this study was slightly lower than previous reports from endemic regions of Spain, likely due to the inclusion of samples from non-endemic areas (Villanueva-Saz et al. 2022b). Although studied ferrets received macrocyclic lactones, these were for other parasitic preventions rather than specific *D. immitis* prophylaxis. Although *D. immitis is* transmitted by infected mosquitoes, seropositivity was notably higher among indoor ferrets (7.03%). One potential explanation for is that indoor environments may inadvertently foster conditions conducive to vector proliferation—such as vegetation and artificial water sources—that facilitate the presence of mosquitoes carrying microfilariae (Villanueva-Saz et al. 2022a, b, c).

The limitations of this study include that serology indicates previous exposure to these three parasites, but they do not confirm clinical susceptibility. Moreover, the retrospective design, uneven sampling distribution, and limited regional data, require cautious interpretation. The study strengths include its large sample size and extended study period, providing the first epidemiological insights into *L. infantum*, *D. immitis*, and *T. gondii* in ferrets in Spain. This survey represents the first cross-sectional study on ferret exposure to *L. infantum*, *T. gondii*, and *D. immitis* in any country. Future research should investigate coinfections in ferrets with clinical leishmaniasis and incorporate molecular assays, such as PCR, to accurately determine their infection status. This approach would provide valuable insights to better establish the epidemiological role of infected ferrets.

In summary, this is the first study to examine the seroprevalence of antibodies against *L. infantum*, *T. gondii*, and *D. immitis* in ferrets across different regions of Spain. Our findings indicate that ferrets in these areas are exposed to these three parasites. Given these results, we recommend the use of preventative measures, including annual screening for *L. infantum* through a validated quantitative serological test for early detection of seropositive status, and the administration of macrocyclic lactones to prevent *D. immitis* infection. Further research is needed to assess infection rates of *L. infantum*, *D. immitis*, and *T. gondii* in ferrets in other endemic regions of Europe.

## Supplementary Information

Below is the link to the electronic supplementary material.

Supplementary Material 1

## Data Availability

The data that support the findings of this study are available from the corresponding author upon request.
