# Supplementary material for: Seroepidemiological study of Leishmania infantum, Toxoplasma gondii and Dirofilaria immitis in pet ferrets (Mustela putorius furo) in Spain
Source: Vet Res Commun. 2025 Apr 7;49(3):160. doi: 10.1007/s11259-025-10729-5 (PMC11976796; doi:10.1007/s11259-025-10729-5)
Supplement: Supplementary file 1 — Supplementary Material 1 [file 11259_2025_10729_MOESM1_ESM.docx]

| Parasite | Serology | | Age | | *p* | Gender | | *p* | Neutering | | *p* | Cohabitation | | *p* | Other clinical conditions | | *p* | Lifestyle | | *p* |
| --- | --- | --- | --- | --- | --- | --- | --- | --- | --- | --- | --- | --- | --- | --- | --- | --- | --- | --- | --- | --- |
| *L. infantum* | Positive | 10.5% (n=47) | Juvenile  Adult  Senior | 0.5% (n=2)  4.4% (n=18)  6.4% (n=26) | 0.052 | Male  Female | 5.9% (n=24)  5.4% (n=22) | 0.754 | Intact  Hormone implanted  Surgically neutered | 2.5% (n=10)  4.2% (n=17)  4.7% (n=19) | 0.057 | Yes  No | 8.9% (n=33)  3.3% (n=12) | 0.861 | Yes  No | 9.4% (n=38)  2% (n=8) | 0.02 | Indoor  Mixed | 8.3% (n=33)  3.2% (n=13) | 0.326 |
|  | Negative | 89.5% (n=401) | Juvenile  Adult  Senior | 15.5% (n=63)  34.6% (n=141)  38.6% (n=157) |  | Male  Female | 48.9% (n=200)  39.8% (n=163) |  | Intact  Hormone implanted  Surgically neutered | 29.7% (n=120)  36.4% (n=147)  22.5% (n=91) |  | Yes  No | 61.2% (n=227)  25.9% (n=95) |  | Yes  No | 49.1% (n=199)  39.5% (n=160) |  | Indoor  Mixed | 55.9% (n=223)  32.6% (n=130) |  |
| *T. gondii* | Positive | 2.7% (n=12) | Juvenile  Adult  Senior | 0%  (n=0)  0.5% (n=2)  1.7% (n=7) | 0.113 | Male  Female | 1% (n=4)  1.2% (n=5) | 0.737 | Intact  Hormone implanted  Surgically neutered | 1% (n=4)  0.7% (n=3)  0.5% (n=2) | 0.718 | Yes  No | 1.3% (n=5)  0.8% (n=3) | 0.6 | Yes  No | 1.7% (n=7)  0.5% (n=2) | 0.225 | Indoor  Mixed | 1% (n=4)  1.2% (n=5) | 0.292 |
|  | Negative | 97.3% (n=436) | Juvenile  Adult  Senior | 15.8% (n=65)  38.8% (n=159)  43.2% (n=177) |  | Male  Female | 53.6% (n=221)  44.2% (n=182) |  | Intact  Hormone implanted  Surgically neutered | 31% (n=126)  40% (n=163)  26.8% (n=109) |  | Yes  No | 69.5%  (n=257)  28.4% (n=105) |  | Yes  No | 57.1% (n=233)  40.7% (n=166) |  | Indoor  Mixed | 63.2% (n=254)  34.6% (n=139) |  |
| *D. immitis* | Positive | 10.3% (n=46) | Juvenile  Adult  Senior | 2% (n=8)  4.5% (n=18)  4.7% (n=19) | 0.9 | Male  Female | 6.2% (n=25)  5% (n=20) | 0.999 | Intact  Hormone implanted  Surgically neutered | 2.7% (n=11)  6.3% (n=25)  2.2% (n=9) | 0.103 | Yes  No | 8% (n=29)  3.6% (n=13) | 0.722 | Yes  No | 6.2% (n=25)  5% (n=20) | 0.748 | Indoor  Mixed | 6.6% (n=26)  4.3% (n=17) | 0.737 |
|  | Negative | 89.7 (n=402) | Juvenile  Adult  Senior | 13.9% (n=56)  34.6% (n=139)  40.3% (n=162) |  | Male  Female | 48.3% (n=195)  40.5% (n=164) |  | Intact  Hormone implanted  Surgically neutered | 29.6% (n=118)  34.6% (n=138)  24.6% (n=98) |  | Yes  No | 63.1% (n=229)  25.3% (n=92) |  | Yes  No | 52.3% (n=209)  36.5% (n=146) |  | Indoor  Mixed | 57.1% (n=225)  32% (n=126) |  |

**Supplementary Table S1.** Correlation of seroprevalence of *Leishmania infantum*, *Toxoplasma gondii* and *Dirofilaria immitis* with clinical factors.
